# Peripheral blood cells and immune checkpoint inhibitor-associated myocarditis: a retrospective clinical study with pharmacovigilance and single-cell analysis

**DOI:** 10.3389/fimmu.2026.1834913

**Published:** 2026-08-26

**Authors:** Zhenli Li, Jing He, Zipei Ma, Piaoran Liu, Zhengkun Guan, Shaoyan Du, Tiezhu Yao, Guang Liu, Jing Liu, Ling Guo, Yaozhong Zhang, Tenghui Wang, Mengjia Li, Jingtao Ma

**Affiliations:** 1 Department of Cardiology, The Fourth Hospital of Hebei Medical University/The Tumor Hospital of Hebei Province, Shijiazhuang, Hebei, China; 2 Hebei Medical University, Shijiazhuang, Hebei, China; 3 Department of Cardiology, Peking University International Hospital, Beijing, China

**Keywords:** biomarkers, FDA adverse event reporting system (FAERS), immune checkpoint inhibitor, myocarditis, nomogram, prognosis, single-cell analysis

## Abstract

**Background:**

Immune checkpoint inhibitor (ICI)-associated myocarditis (ICI-M) is a rare but potentially lethal immune-related adverse event (irAE). Early identification of fulminant (severe) cases remains challenging, and the peripheral immune derangements underlying ICI-M are incompletely characterized.

**Methods:**

The disproportionality analysis was performed using the FDA Adverse Event Reporting System (FAERS) database (2015–2026) for patients treated with eight ICIs. A retrospective cohort of 100 patients who developed ICI-M was stratified into severe and non-severe groups. Peripheral blood cells and their derived ratio index were analyzed. A severity-predictive nomogram was developed using features selected by three machine learning methods, and was evaluated by calibration, receiver operating characteristic, and decision curve analyses. Single-cell RNA-seq data from peripheral blood mononuclear cells were analyzed to explore immune landscape alterations for ICI-M.

**Results:**

FAERS analysis revealed strong signals for ICI-M across all eight ICIs, with >80% of cases occurring within three months of therapy. Associations between peripheral blood cells/derived ratio indexes and ICI-M were identified: the (neutrophil + monocyte) to lymphocyte ratio (NMLR), cTnI, and NT-proBNP were identified as key predictors of severe ICI-M. The 3-feature-based exploratory nomogram demonstrated a relatively good predictive performance (Area under curve = 0.849) with internal validation performed via 1000 bootstrap samples. Single-cell profiling identified monocyte expansion and lymphocyte contraction for ICI-M and its severity. The pseudotime ordering, ligand–receptor inference, subcluster differential abundance analysis, and pathway enrichment analyses associated a distinct *FCGR3A* + monocyte subset with a potential role in ICI-M development.

**Conclusion:**

This study provides a multi-dimensional view of ICI-M, integrating clinical and single-cell immune profiling analysis of peripheral blood cells as well as pharmacovigilance data. The developed nomogram may serve as a potential tool for identifying severe ICI-M, although external validation in independent cohorts is warranted. Insight into the immune landscape may inform future therapeutic strategies targeting specific cell–cell interactions in ICI-M.

## 1 Introduction

Immune checkpoint inhibitors (ICIs) have revolutionized the treatment landscape of numerous malignancies by selectively blocking of negative immune regulatory molecules, including cytotoxic T-lymphocyte-associated protein 4 (CTLA-4) and the programmed death 1 (PD-1)/programmed death-ligand 1 (PD-L1) (1). However, the ICI-induced non-selective activation of the immune system, while conferring its significant antitumor efficacy, has precipitated a spectrum of immune-related adverse events (irAEs) (2, 3). Immune checkpoint inhibitor-related myocarditis (ICI-M), known as a rare (incidence ranging from 0.27 to 1.14%) but lethal irAEs (4), exhibited a striking overall mortality risk of 30%-50% (5), the presentations of which can range from mild, asymptomatic troponin elevation to life-threatening. Previously, the signals of ICI-M have been detected using the databases named VigiBase and FDA Adverse Event Reporting System (FAERS) (6, 7). However, to accurately reflect the current safety landscape, incorporating the most recent quarterly data updates is indispensable. Moreover, FAERS provides severity-related variables and time-to-onset data for ICI-M, enabling comparisons with our real-world clinical cohort.

As objective and easily accessible numerical data, peripheral blood cells and their derived indexes are more amenable to clinical application. Several studies have demonstrated the association between per blood cell (especially for lymphocytes and monocytes) and ICI-M, both in terms of disease occurrence and severity (8–10). Additionally, previous studies have linked blood cell-derived inflammatory ratios to the severity of ICI-M and other myocarditis, such as the systemic inflammation response index (SIRI) and systemic immune-inflammation index (SII) (11, 12). Additionally, single-cell analyses, regarding peripheral blood mononuclear cells (PBMCs) from ICI-M patients, have also been conducted for quantitative shifts in the proportional abundance of distinct immune subsets to gain a deeper understanding of the ICI-M landscape at the cellular level (13, 14). However, there is still a critical need for further clinical analysis of the above association integrated with a single-cell perspective. More fatal adverse cardiovascular events were observed in the severe ICI-M group (5, 15), highlighting the clinical significance of identifying severe ICI-M patients. Predictive risk factors have been explored for the occurrence of severe ICI-M, such as elevated troponin and NT-proBNP, new decline in left ventricular ejection fraction (LVEF), and abnormal electrocardiogram (ECG) parameters (15–17). Nevertheless, the potential role of peripheral blood cells combined with these reported cardiac parameters to predict ICI-M severe is extremely research-worthy.

**Figure 1:**
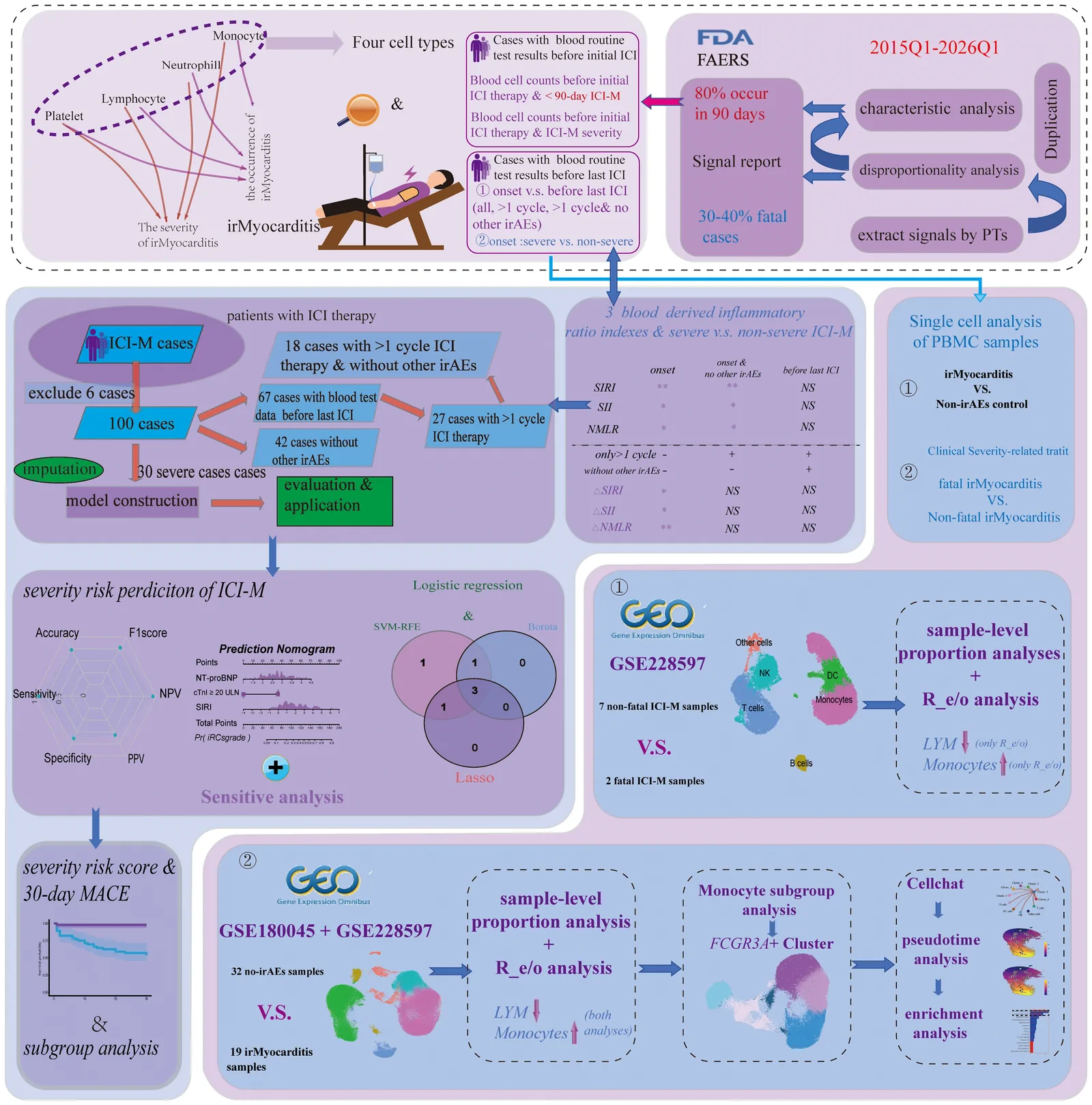
The overall design of the present study.

Relying on previous studies, we formulated a central hypothesis: peripheral blood immune shifts play a potential role in the pathophysiological features of ICI-M that not only correlate with the occurrence and severity of disease but also reflect the underlying inflammatory mechanisms, with the aim of providing viable targets for both clinical risk stratification and mechanistic understanding of ICI-M. This study aimed (1) to capture the current pharmacovigilance landscape of ICI-M through the FAERS database compared with our clinical data; (2) to identify associations between peripheral blood cells/blood-derived ratio indexes and ICI-M; (3) to establish a severity-predictive model by integrating core blood-derived ratio indexes and cardiac biomarkers; and (4) to conduct a discovery-oriented single-cell analysis of PBMCs for ICI-M to characterize the potential immune microenvironment signatures, which may provide exploratory insights into the cellular underpinnings of the hematological parameters associated with ICI-M. By integrating pharmacovigilance data, clinical indicators, and single-cell resolution (**Figure 1**), this study sought to provide a multidimensional immunological landscape with potential clinical application of peripheral blood cells for ICI-M.

## 2 Materials and methods

### 2.1 FAERS data processing and signal mining

We performed a pharmacovigilance analysis of adverse events associated with ICIs using data from the FAERS. Reports involving eight ICIs (cemiplimab, nivolumab, pembrolizumab, atezolizumab, avelumab, durvalumab, ipilimumab, and tremelimumab) were extracted from the FAERS public dashboard for the period spanning Q1 2015 to Q1 2026. Following FDA-recommended data cleaning steps, duplicate entries were removed to enhance data reliability as much as possible. Adverse event cases related to the included ICIs were identified using the Medical Dictionary for Regulatory Activities (MedDRA v.26.0) and were initially defined based on the preferred terms (PTs) “MYOCARDITIS” and “IMMUNE-MEDIATED MYOCARDITIS”. Moreover, we also utilized a series of PTs for ICI-M defined by Gougis et al. (6) for a more generalized analysis. Extracted variables comprised patient demographics, report countries, reporter types, medication information, adverse reaction type, severity-related features, and clinical outcomes.

**Figure 2:**
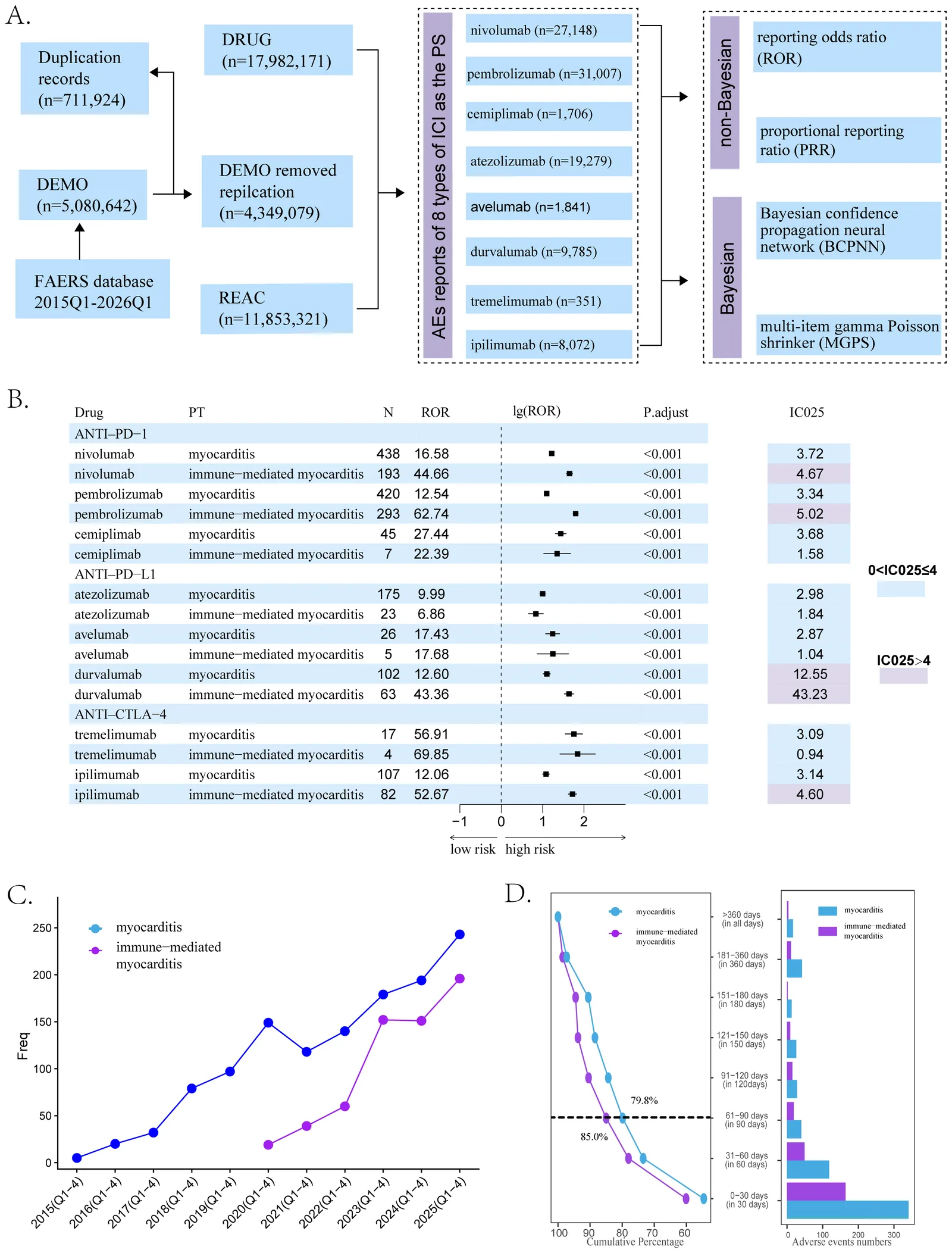
Characteristics of Immune checkpoint inhibitor-related myocarditis (ICI-M) [preferred terms (PTs): MYOCARDITIS and IMMUNE-MEDIATED MYOCARDITIS] associated with 8 types of immune checkpoint inhibitors (ICIs). **(A)** FDA Adverse Event Reporting System (FAERS) Data analysis workflow. **(B)** Reporting odds ratios (ROR) and information component (IC) for 8 types of ICIs in relation to ICI-M. **(C)** Overall numbers of ICI-M events reported per year for all 8 types of ICIs. **(D)** Onset time from initial ICI therapy and its cumulative percentage of all 8 types of ICIs associated ICI-M at different time points (only reports that provided the onset time were included).

To evaluate the strength of potential safety signals, we computed commonly used disproportionality measures, including the reporting odds ratio (ROR), proportional reporting ratio (PRR), empirical Bayesian geometric mean (EBGM), and information component (IC) (18). Formulas and interpretations and the criteria for signal detection for these metrics are provided in **Supplementary Table S1**. Statistically significant signals were further examined with chi-square tests, and the Benjamini–Hochberg procedure was applied to control the false discovery rate in multiple testing; an adjusted p-value< 0.05 was considered significant. The onset time of ICI-M was extracted as the interval between EVENT_DT (date of adverse events (AE) occurrence) and START_DT (date of ICI initiation) after excluding reports with data input errors or inaccurate date. We further displayed the distribution of onset time for ICI-M and the number of AEs in different years. The entire data extraction and analytical workflow adhered to established pharmacovigilance methodology and standard operating procedures, as outlined in **Figure 2A**.

### 2.2 Clinical data analysis

#### 2.2.1 Study population

Patients who received immunotherapy and were clinically diagnosed with ICI-M, at the Fourth Hospital of Hebei Medical University between June 2021 and December 2025, were enrolled in this study. All patients were diagnosed with various types of cancers and received at least one cycle of ICI therapy. Exclusion criteria are as follows: (1) the presence of severe infections when collecting blood samples; (2) receiving blood parameter-altering medication within a week prior to blood sample collecting; (3) poor treatment adherence; (4) missing data at ICI-M onset (with more than 20% of the core covariates missing). Diagnosis of ICI-M was based on major and minor clinical criteria defined by the 2022 European Society of Cardiology (ESC) Guidelines on cardio-oncology (19), with definite/probable/possible diagnostic classification by Bonaca’s diagnostic criteria (20). The details of the diagnostic criteria can be found in **Supplementary Table 2**. According to International Cardio-Oncology Society (IC-OS) consensus statement (19, 21), patients were further stratified into severe (fulminant) and non-severe (non-fulminant) cohorts. Severe ICI-M was defined if a patient met at least one major clinical criterion (15, 17): (1) hemodynamic instability; (2) heart failure requiring non-invasive or invasive ventilation; (3) complete or high-grade heart block; (4) significant ventricular arrhythmia. Patients who met none of the above criteria were classified as the non-severe group. All diagnosed cases were reconfirmed and stratified by two cardio-oncology specialists, named Guang Liu and Tiezhu Yao, with differential diagnosis performed against acute coronary syndrome, Takotsubo cardiomyopathy, and other cancer therapy-related cardiac toxicities to reduce the diagnostic bias (**Supplementary Tables 3**, **7**). The studies involving humans were approved by the Ethics Committee of the Fourth Hospital of Hebei Medical University. The studies were conducted in accordance with the local legislation and institutional requirements. Written informed consent for participation was not required from the participants or the participants’ legal guardians/next of kin in accordance with the national legislation and institutional requirements. The ethics committee/institutional review board waived the requirement of written informed consent for participation because this was a retrospective study with no additional intervention for the patients.

Baseline clinical characteristics, routine blood test results, cardiac biomarkers [cardiac troponin I (cTn I), N-terminal probrain natriuretic peptide (NT-proBNP), creatine kinase myocardial band (CK), creatine kinase myocardial band (CKMB), lactate dehydrogenase (LDH)], and echocardiographic parameters such as LVEF of ICI-M patients at onset were collected. Baseline clinical characteristics involved cancer types, stage grades of cancers, past treatments of cancers, cardiovascular risk factors, medical comorbidities, comprehensive details regarding immunotherapy and immunosuppressive therapy for ICI-M. Moreover, routine blood test results at before initial ICI therapy and before the within one week of the last ICI therapy were also recorded. Absolute neutrophil (NE), absolute monocyte (MO), absolute lymphocyte (LYM), platelet (PLT) counts were also collected from blood routine tests. Beyond standard laboratory tests, this study investigated the association between a set of blood cell-derived composite biomarkers, and the severity of ICI-M. Patients were followed up for 30 days after ICI-M onset. The primary endpoint of follow-up was the occurrence of the major adverse cardiovascular events (MACEs) at the 30-day follow up. The MACEs are defined as follows (5): (1) ventricular tachyarrhythmias (including non-sustained episodes); (2) conduction abnormalities (second- or third-degree atrioventricular block) necessitating pacemaker implantation, (3) decompensated heart failure requiring hospitalization; (4) sudden cardiac death. Time to MACE was defined as the interval (days) from ICI-M diagnosis to the first onset of a MACE. The study data were censored at the end of the 30-day follow-up period or at the time of loss to follow-up, whichever came first. The overall study follow-up cutoff date was January 10, 2026.

#### 2.2.2 Analysis of the association between peripheral blood cells and ICI-M

First, we investigated the differences of blood cells between ICI-M at onset and before last ICI therapy (without ICI-M status). The paired Wilcoxon signed-rank test was used to compare the LYM, MO and LYM to MO ratio (LMR) at onset and before the last cycle of ICI therapy for all ICI-M patients with completed data; and subgroup analyses, including ICI-M patients receiving >1 cycle of ICI therapy and those receiving >1 cycle of ICI therapy without other irAEs, were performed. Second, because several studies have reported an occurrence rate of over 70% for ICI-M in initial 3 months (6, 22), we further explored the association between NE, LYM, MO, and PLT before initial ICI therapy and ICI-M occurring in 3 months after initial treatment. A comparison of NE, LYM, MO, and PLT before initial ICI therapy between severe and non-severe ICI-M groups was also performed. Third, NE, LYM, MO, and PLT at ICI-M onset were also compared between the severe and non-severe groups with subgroup analyses. Comparisons between two independent groups were performed using the Mann–Whitney U test in the above analyses.

#### 2.2.3 The association between blood cell-derived ratio indexes and severity of ICI-M

Based on previously established formulas, we calculated the following 3 inflammatory ratio indexes: SIRI = NE × MO/LYM, SII = NE × PLT/LY, (neutrophil + monocyte) to lymphocyte ratio (NMLR) = (NE + MO)/LYM. The formula describing the change rates of ratio indexes from the status before last ICI therapy to that at onset (ΔRatio index) is as follows:


$$\Delta \text{Ratio index}=\frac{\text{Ratio indexonset}-\text{Ratio indexbefore last ICI}}{\text{Ratio indexbefore last ICI }}$$

To better display and allow for simpler model fitting and interpretation, the inflammatory ratio indexes and cardiac parameters all underwent a logarithmic transformation or were categorized into ordinal variables. The details can be found in **Supplementary Table 4**. We comprehensively compared the distribution of the above 3 ratio indexes, as well as 3 ΔRatio index, at onset and before the last ICI therapy between the severe and non-severe groups utilizing Wilcoxon signed-rank test. Spearman’s correlation test was used to determine the correlation between 3 ratio indexes/ΔRatio index and the severity of ICI-M. For each ΔRatio index, univariate logistic regression was performed to evaluate its potential risk for patients of developing high-grade ICI-M. The paired Wilcoxon signed-rank test was also used to compare the ratio indexes at onset and before the last ICI therapy. Furthermore, subgroup analyses for each ratio index and ΔRatio index were also performed by a step-by-step exclusion of patients who received only one cycle ICI therapy and those with other non-myocarditis irAEs.

#### 2.2.4 Nomogram construction and evaluation for identifying severe ICI-M

To handle missing data at the time of ICI-M onset, multiple imputation was performed using the “mice” package (v.3.19.0) using the predictive mean matching method (m = 5). Based on the results, we preliminarily filtered out features with significance by univariate logistic regression (LR). Subsequently, to identify key traits, nine candidate features were further analyzed to obtain the most significant features using the Least Absolute Shrinkage and Selection Operator (LASSO), the Support Vector Machine -Recursive Feature Elimination (SVM-RFE) algorithm, and the Boruta algorithm. The intersection of the results from these three algorithms was used to identify key features for further investigation. Using these key features, a model was established based on LR, and a nomogram was developed. Moreover, several methods, with bootstrap with 1000 repetitions, were employed to evaluate and validate the performance of the nomogram, including calibration curves, the area under the receiver operating characteristic curves (AUCs), and decision curve analysis (DCA). An additional five metrics for assessing the performance of the established models were also evaluated, including accuracy, sensitivity (SE), specificity (SP), positive predictive value (PPV), and negative predictive value (NPV) and accuracy. To evaluate whether the combined nomogram exhibited better predictive performance than single predictors, DeLong tests were applied to compare the AUC of the nomogram against the AUC of each of its three individual predictive variables. To further assess the stability of our model, we conducted subgroup sensitivity analysis. The predicted probability derived from the nomogram was treated as a composite risk variable, and its association with the outcome was separately tested across a series of clinical subgroups. Finally, we developed a web-based online risk calculator, which not only allows real-time risk estimation for ICI-M but also allows independent external validation. During the above process, several key R packages were used, including “glmnet” (v.4.1-10), “e1071” (v.1.7-17), “caret” (v.7.0-1), “Boruta” (v.9.0.0), “pROC” (v.1.19.0.1), “fbroc” (v.0.4.1) and”shiny”(v.1.13.0).

Based on the established nomogram, we calculated the severity risk score of each patient using the package “nomogramFormula” (v.1.2.0.0). We also explored the potential of the risk scores to stratify risk for ICI-M and the development of 30-day MACEs with subgroup analysis. Using the “surv_cutpoint” function, we extracted an optimal threshold to stratify ICI-M patients into two prognostic groups, the low-risk group and high-risk group (23). Kaplan–Meier (K–M) curves were also plotted to compare and illustrate the 30-MACE survival of high- and low-risk groups, followed by subgroup analyses. Finally, a web-based online risk calculator was developed for further potential clinical use, which was also an exploratory implementation tool and a platform to facilitate future external validation.

### 2.3 Single-cell RNA-seq analysis

#### 2.3.1 Sample selection

PBMC samples of individuals receiving ICI therapy were extracted from the Gene Expression Omnibus (GEO) databases (access ID: GSE180045 and GSE228597, details in **Supplementary Table 5**) for single-cell RNA-seq analysis (13, 14). After excluding samples from patients who had received steroid therapy prior to blood collection, we finally included 32 samples (29 from GSE228597 and 3 from GSE180045) receiving ICI therapy without any irAEs as the control group and 19 samples (17 from GSE228597 and 2 from GSE180045) with ICI-M as the disease group. The meta data of the above samples can be found in **Supplementary Table 5**. Among the 17 samples from the GSE228597 dataset, the clinical severity-related information for ICI-M patients classified as “Fatal due to myocarditis,” were available to explore the association between PBMC sub-cell groups and the ICI-M severity. Two samples from fatal cases were selected as the severe group. To mitigate potential bias arising from the small group size of fatal ICI-M, the non-fatal samples were then refined by selecting only those (i) with biopsy or autopsy diagnosis and (ii) with myocarditis onset occurring within 90 days of the initial ICI dose. This selection process yielded seven non-fatal ICI-M samples for comparative analysis with the fatal group. The corresponding metadata and sample details are listed in **Supplementary Table 5**.

#### 2.3.2 Quality control, integration and clustering

Data were processed using the “Seurat” package (v.5.4.0). Single-cell RNA sequencing data underwent standard quality control procedures. Cells were filtered based on the following criteria: (1) cells expressing between 200 and 5,000 genes; (2) total Unique Molecular Identifier (UMI) counts<25,000; (3) mitochondrial gene content<20%. DoubletFinder (v.2.0.6) was used to identify and remove doublets based on simulated doublet profiles, where each cell receives a doublet score and cells exceeding the threshold were excluded from downstream analysis. Normalization was performed using “NormalizeData” function, followed by the selection of the top 2000 highly variable genes and data scaling. Principal component analysis (PCA) was utilized to perform dimensionality reduction, with batch effect corrected across individual samples using the “RunHarmony” function (dims = 1:15). Uniform Manifold Approximation and Projection (UMAP) clustering were applied using the first 15 principal components with different resolutions (0.4 for PBMC cell groups). For the process of monocyte reclassification, cells annotated as monocytes were extracted and reclustered using the same parameters as PBMC cell clustering.

#### 2.3.3 Cell cluster annotation and cell proportion comparison

For the annotation of PBMC clusters, we performed differential expression analysis across all clusters using Seurat’s “FindAllMarkers” function. The top 20 markers for each cluster were initially used as input to assist cell type annotation on the ACT web server (http://xteam.xbio.top/ACT/). Based on the automated annotation, we further validated the results using gene markers for different cell types as in the study by Xiao et al. (24). As for dendritic cells (DCs), *FCER1A*, *CLEC9A*, and *CLEC4C* served as marker genes. To assess group enrichment preference within each cell subgroup, we computed the Ro/e (observed-to-expected ratio, expected counts derived from chi-square contingency, >1 means enrichment,<1 means depletion) statistic based on chi-square derived expected cell counts (25, 26). To compare cell proportions between the ICI-M group and the non-irAEs control group, a two-sided Wilcoxon rank-sum test was applied across all samples, and differences with p< 0.05 were considered statistically significant.

#### 2.3.4 Cell–cell communication and pseudotime trajectory analysis

Cell–cell communication was analyzed with the “CellChat” package (v.2.2.0) for annotated cell types, which integrates single-cell transcriptomic data to quantify ligand–receptor pairs and identify key regulatory units in signaling networks. Cellular differentiation trajectories for MOs were reconstructed using the “Monocle3” package (v.1.4.26) based on pseudotime ordering of single-cell transcriptomic profiles. This method enables the inference of developmental lineages, identification of branch points associated with cell fate decisions, and visualization of gene expression dynamics along pseudotime. The plot_genes_in_pseudotime function was used to visualize the expression dynamics of selected genes along the pseudotime trajectory and to show how gene expression levels change continuously as cells progress through differentiation. The “FeaturePlot” function was used to visualize the expression of selected genes across the low-dimensional embedding, where each cell is colored according to the normalized expression intensity of the gene of interest.

#### 2.3.5 Pathway enrichment analysis and transcription factor activity analysis

To measure differential gene expression, the “FindMarkers” function was used with the default Wilcoxon rank-sum test method. Only genes with logFC > 0.5 and adjust.p<0.05 were used. Pathway analysis of the selected genes was performed using the R packages “Enrichr” (v.3.4) based on MSigDB Hallmark 2020 database. Pathways were selected to display when the adjust.p was<0.05. Gene set enrichment analysis (GSEA) was performed on the preranked gene list based on log2fold changes from inter-group (ICI-M vs. control) comparisons, and enrichment was assessed against the Hallmark gene set collection (h.all.v2026.1.Hs.symbols) using the “clusterProfiler” package (v.4.18.2) and its “GSEA” function. Gene sets were filtered to include only those containing 10–500 genes. Enrichment scores with adjust.p-value<0.05 were considered significant. PROGENy pathway activity analysis was performed on the normalized gene expression matrix using the “PROGENy” package (v.1.28.0). Activity scores for 14 canonical signaling pathways were estimated based on precomputed gene weights, with default parameters applied. Transcription factor (TF) regulatory networks were constructed using “pySCENIC” (v.0.12.1) based on “python” (v.3.8.1) (27). The “GRNBoost2” algorithm was used to infer co-expression networks, and CisTarget was employed to identify direct transcriptional targets and binding motifs. Regulon activity in each cell was quantified using AUCell. Cluster-specific regulons were identified based on the regulon specificity score (RSS), and their activity patterns were visualized.

### 2.4 Statistical analysis

Besides the methods described above, baseline characteristics of ICI-M patients were described as follows: (1) continuous variables were reported as mean with standard deviation or median with interquartile range (IQR) and were compared using the t-test or Mann–Whitney U test; (2) Categorical variables were presented as numbers with percentages, and comparisons were performed using the Chi-squared test or Fisher’s exact test. Statistical analysis was performed using R software (v.4.4.1) and Python (v.3.8.1), with the significance level set at p<0.05 based on a two-sided test.

## 3 Result

### 3.1 Signal mining results of ICI-associated myocarditis using FAERS

Utilizing the pharmacovigilance data from the FAERS database, we mainly focused on ICI-related AE reported from the first quarters of 2015 to 2026 (**Figure 2A**). All of the 8 ICIs analyzed in the study showed significant signals for myocarditis with a minimum ROR of 6.20 and a minimum IC025 of 0.94 (**Figure 2B**; **Supplementary Figure 1A**). As for the more specific PT, immune-mediated myocarditis, a significant association was also observed with all 8 ICIs (**Figure 2B**). Meanwhile, as shown in **Figure 2C**, the number of reported AEs with ICIs as the primarily suspected drugs seemed to show a rising pattern over the past 5 years. In terms of the time-to-onset of the myocarditis/immune-mediated myocarditis (with available data), nearly 80% of the events emerged in the initial 3 months following the ICI therapy and over a half emerged in the initial 30 days following the ICI therapy (**Figure 2D**). Additionally, according to myocarditis defined by Gougis et al., a similar tendency was observed with a 79.8% event occurrence within the initial 3 months following ICI therapy (**Supplementary Figures 1B, C**). The characteristics of cases used in analysis from FAERS database are presented in **Supplementary Table 6**. Over 30% of the reported cases developed fatal ICI-M and most were serious cases with poor clinical outcomes.

### 3.2 Characteristics of ICI-M patients

As shown in **Table 1**, 100 patients diagnosed with ICI-M, including 70 non-severe and 30 severe ICI-M patients (**Supplementary Figure 2F**), were enrolled in this study, among which 24 (24.0%) patients were classified as definite ICI-M, 22 (22.0%) as probable ICI-M, and 54 (54.0%) as possible ICI-M. Cardiac magnetic resonance (CMR) was performed in 39 patients, among which 21 (53.8%) presented confirmed CMR changes for myocarditis, 12 (30.8%) had suggestive CMR changes for myocarditis, and 6 (15.4%) had negative CMR changes for myocarditis (**Supplementary Table 7**). Differences between the non-severe and severe ICI-M cohorts were evaluated. The results showed no significant difference of basic information among the two cohorts, including age, sex, body mass index (BMI), smoking history and drinking history. As for cancer-related variables, over 70% of patients were in tumor stage IV and over 60% were diagnosed with gastrointestinal tumors in the two cohorts. Regarding previous history of tumor therapy, no significant difference with regard to chemotherapy, targeted therapy, radiotherapy, and surgery were found. Additionally, cardiac related blood parameters at ICI-M onset in severe group were significantly higher compared with the non-severe group, whereas no significant differences in LVEF were observed. Regarding immunosuppressive therapy, the severe group received higher doses of corticosteroid therapy and more additional immunosuppressive treatments in our study. Among the severe cases, 6 (20.0%) patients underwent temporary pacemaker implantation, and only one patient agreed to receive extracorporeal membrane oxygenation (ECMO) support. Comorbidities between the two cohorts also displayed no significant differences (**Supplementary Figure 2A**). Additionally, we compared the time from initial therapy to onset for each ICI-M and observed over 80% of ICI-M occurred within 90 days from initial therapy (**Supplementary Figure 2B**). In **Supplementary Figures 2C–E**, the details of ICI types, tumor types, non-myocarditis irAEs and clinical symptoms at onset of ICI-M patients are shown. None of the enrolled patients was censored prematurely due to loss to the 30-day follow-up. During this period, the primary composite endpoint (MACE) occurred in 27 ICI-M patients (27.0%).

**Table 1:** The characteristics of immune checkpoint inhibitor-related myocarditis (ICI-M) patients.

Characteristic	Non-severe cohort (N = 70)	Severe cohort (N = 30)	*p*-value
Basic information			
Age	68 (62, 72)	70 (63, 74)	0.265
Sex			0.540
Female	23 (32.9%)	8 (26.7%)	
Male	47 (67.1%)	22 (73.3%)	
BMI	24.5 ± 4.0	23.2 ± 2.9	0.061
Smoking history			0.429
No	41 (58.6%)	15 (50.0%)	
Yes	29 (41.4%)	15 (50.0%)	
Drinking history			0.676
No	46 (65.7%)	21 (70.0%)	
Yes	24 (34.3%)	9 (30.0%)	
Cancer-related information			
Bonaca et al.’ criteria			0.059
Definite	13 (18.6%)	11 (36.7%)	
Probable	14 (20.0%)	8 (26.7%)	
Possible	43 (61.4%)	11 (36.7%)	
Tumor type			0.684
Others	25 (35.7%)	12 (40.0%)	
Gastrointestinal tumors	45 (64.3%)	18 (60.0%)	
Tumor stage			0.691
III	19 (27.1%)	7 (23.3%)	
IV	51 (72.9%)	23 (76.7%)	
ICI-related information			
ICI type			0.497
Others	9 (12.9%)	2 (6.7%)	
PD-1 monotherapy	61 (87.1%)	28 (93.3%)	
Prior therapy			
Targeted therapy	23 (32.9%)	5 (16.7%)	0.098
Chemotherapy	55 (78.6%)	26 (86.7%)	0.344
Radiotherapy	25 (35.7%)	9 (30.0%)	0.580
Surgery	22 (31.4%)	10 (33.3%)	0.852
With other irAEs			0.791
No	30 (42.9%)	12 (40.0%)	
Yes	40 (57.1%)	18 (60.0%)	
Cardiac parameters			
cTnI ≥ 20 ULN	25 (35.7%)	23 (76.7%)	<0.001
NT-proBNP (ng/L)	285 (112, 738)	1,230 (480, 5,580)	<0.001
CK ≥ 2000 U/L	16 (22.9%)	14 (46.7%)	0.017
CKMB ≥ 100 U/L	16 (22.9%)	13 (43.3%)	0.039
LDH ≥ 1000 U/L	6 (8.6%)	9 (30.0%)	0.012
LVEF (%)	60 (57, 62)	58 (48, 64)	0.157
Treatment information			
Corticosteroids (mg/day)	80 (40, 120)	500 (200, 500)	<0.001
Other immunosuppressant^a^	17 (24.3%)	15 (50.0%)	0.012

Data are presented as mean ± standard deviation, median [25th–75th percentile] or number (percentage). ^a^Other immunosuppressants include mycophenolate mofetil, anti-thymocyte globulin (anti-CD3antibody), immunoglobulin, plasma exchange, tocilizumab, abatacept (CTLA-4 agonist), alemtuzumab (anti-CD52 antibody), and to facitinib.

### 3.3 Association between peripheral blood cells and ICI-M

As shown in **Supplementary Figure 3A**, a decline in LYM and an increase in MO, were observed in all patients at ICI-M onset compared with the time point before the last ICI therapy. After excluding patients who received only one cycle of ICI, this significant difference disappeared. However, this significance re-emerged after further excluding patients with other irAEs. As for LMR, a significantly lower level was observed in the severe ICI-M group across different subgroups. The relationships between certain types of blood cells before initial ICI therapy and ICI-M (occurring within 90 days and the severity of ICI-M) were evaluated. As shown in **Supplementary Figure 3B**, only MO level before initial ICI therapy was higher in patients who developed ICI-M within 90 days than in those who did not. As shown in **Supplementary Figure 3C**, only LYM levels before initial ICI therapy were significantly higher in the severe group than patients with non-severe ICI-M. Finally, we explored the relationships between certain types of blood cells at ICI-M onset, and the severity of ICI-M. NE, LYM and MO counts were different in the severe and non-severe groups and their subgroups (**Supplementary Figure 3D**). However, PLT counts did not display any differences in all above analysis.

### 3.4 Blood cell-derived ratio indexes and severity of ICI-M

**Figure 3:**
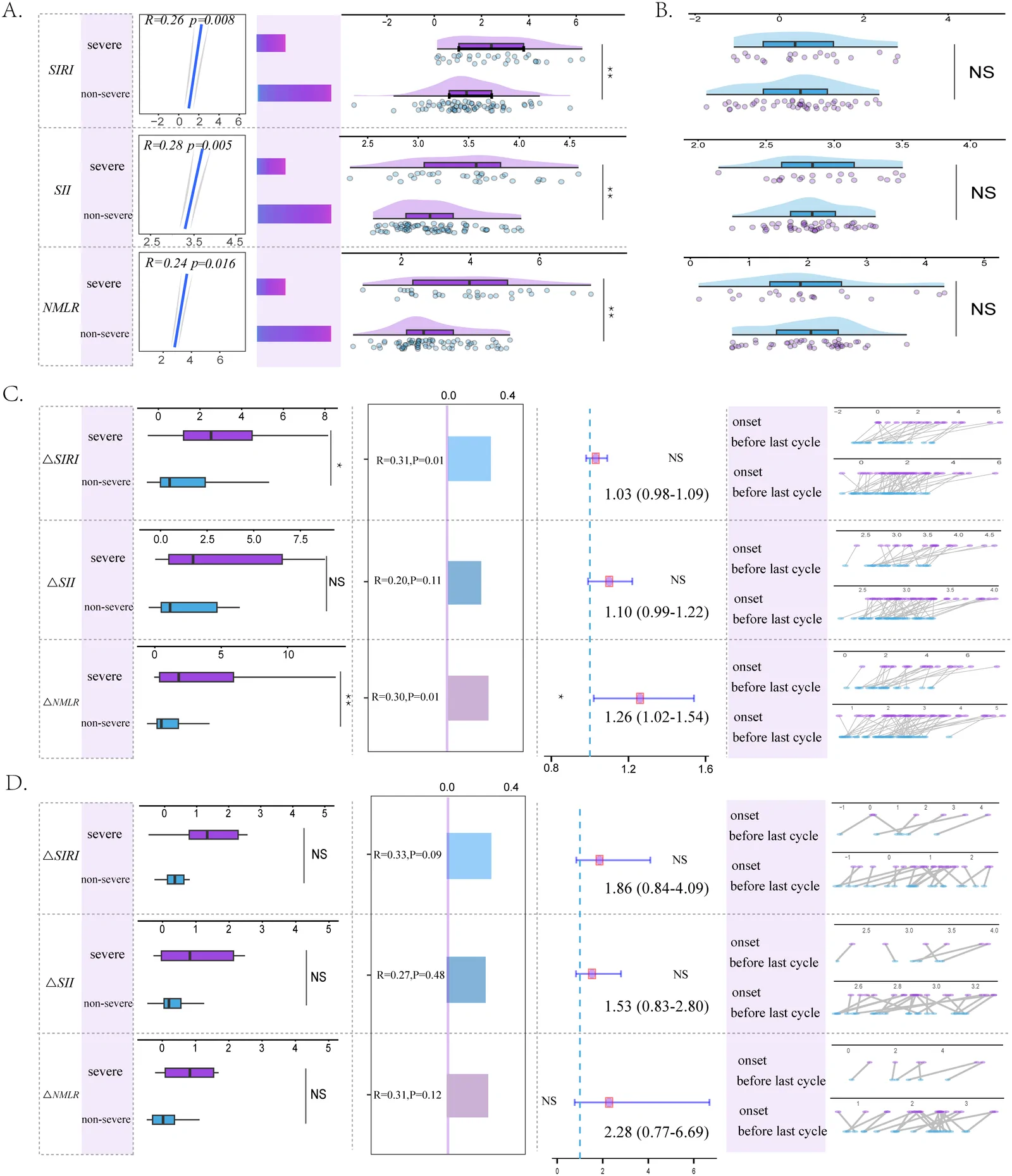
Association of systemic inflammation response index (SIRI), systemic immune-inflammation index (SII), and (neutrophil + monocyte) to lymphocyte ratio (NMLR) with the severity of immune checkpoint inhibitor-related myocarditis (ICI-M). **(A)** Comparison of SIRI, SII, and NMLR levels at the onset of ICI-M between the severe and non-severe groups in all involved ICI-M patients. **(B)** Comparison of these ratio indexes before the last immune checkpoint inhibitor (ICI) therapy between the severe and non-severe groups (including all ICI-M patients with data available). **(C)** Association between ΔSIRI, ΔSII, and ΔNMLR and the severity of ICI-M in all patients with data available. **(D)** Association between ΔSIRI, ΔSII, and ΔNMLR and the severity of ICI-M after excluding patients with only one ICI therapy. *p< 0.05, **p< 0.01; NS, not significant. The values of SIRI, SII, and NMLR were presented after a logarithmic transformation as displayed in **Supplementary Table 4**.

Due to the significant differences in certain blood cell types at the onset in ICI-M, we further explored the relative ratio indexes. SIRI, SII, and NMLR at onset of ICI-M all exhibited a positive correlation with severe ICI-M, with higher levels in the severe group (**Figure 3A**; **Supplementary Figure 4A**). However, no significant differences were observed between the two groups before the last ICI therapy among ICI-M patients, either in all patients or in those receiving more than one cycle ICI therapy (**Figure 3B**; **Supplementary Figure 4B**). Combining the two time points at ICI-M onset and before the last ICI therapy, we further analyzed the association between ΔSIRI, ΔSII, and ΔNMLR, and ICI-M severity. Among all ICI-M patients, both ΔSIRI and ΔNMLR displayed higher levels in the severe group than in the non-severe group, with ΔNMLR owning significant odds ratio (OR) values >1 (**Figure 3C**). However, after excluding patients with only one ICI therapy and those with other irAEs, such significance disappeared (**Figure 3D**; **Supplementary Figure 4C**).

### 3.5 Model construction and short-term MACE survival analysis

**Figure 4:**
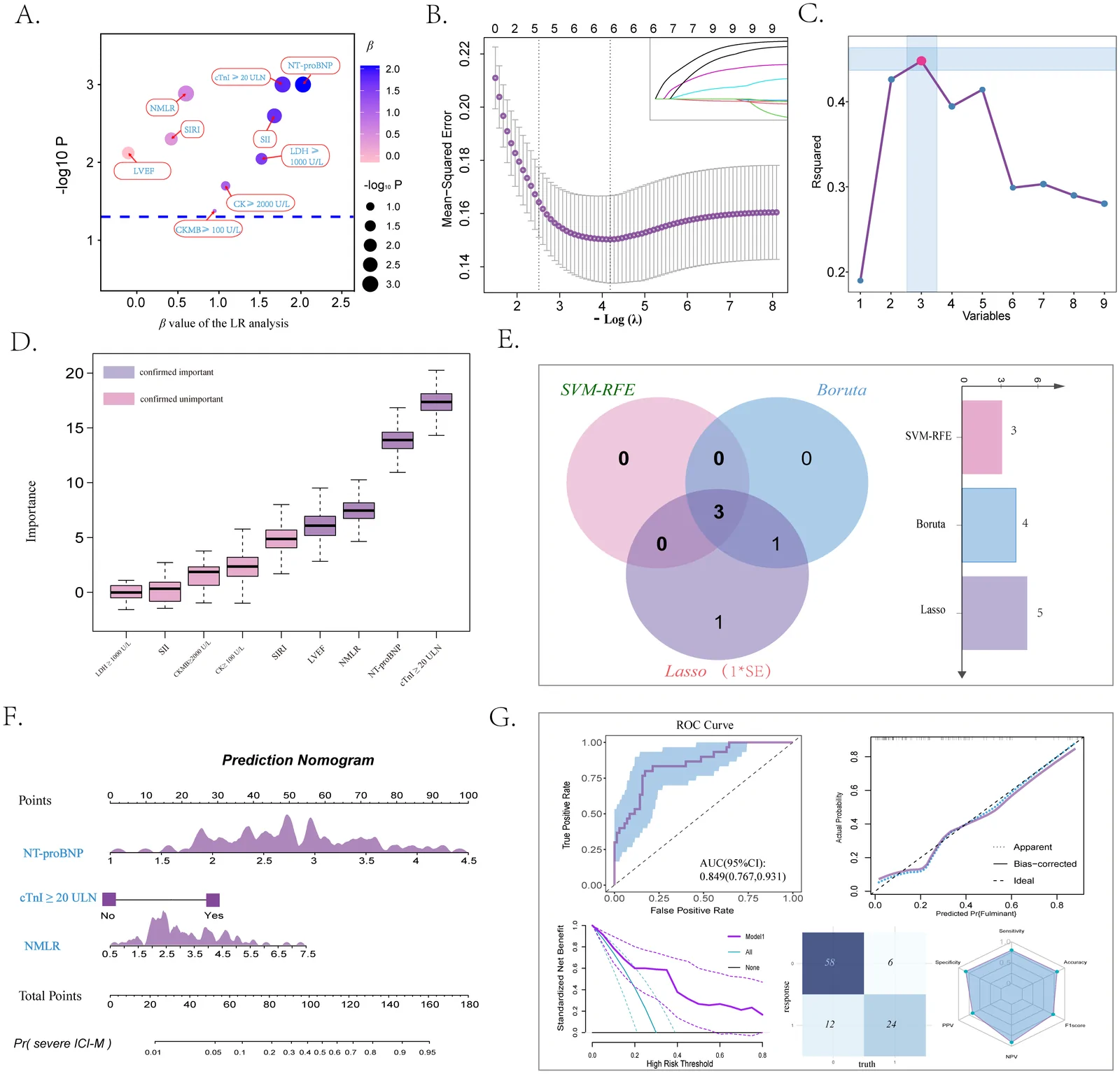
The process of severity-predictive model development and validation. **(A)** Univariate logistic regression analysis identified 9 significant features associated with severe immune checkpoint inhibitor-related myocarditis (ICI-M). **(B–E)** Feature selection using three machine learning algorithms: LASSO **(B)**, Support Vector Machine - Recursive Feature Elimination (SVM-RFE) **(C)**, and Boruta **(D)**, ultimately identified three key predictors: (neutrophil + monocyte) to lymphocyte ratio (NMLR), Troponin I (Tn I), and N-terminal probrain natriuretic peptide (NT-proBNP) **(E)**. **(F)** Nomogram for predicting the probability of high-grade ICI-M based on the three selected features. **(G)** Performance evaluation of the nomogram, including calibration curve, confusion matrix, decision curve analysis for predicting 30-day major adverse cardiovascular events (MACEs), and parameters [sensitivity: 80.0%, specificity: 82.9%, positive predictive value (PPV): 66.7%, negative predictive value (NPV): 90.6%, accuracy: 0.82, F1-score:0.727] for evaluating the performance of the model.

The details of the missing variables before and after imputation can be found in **Supplementary Table 8**. The 3 ratio indexes with 6 cardiac-related indicators were selected for further analyses, rather than the 3 change rates of these indexes described in section 3.5. Initially, we conducted univariate LR analysis and found all features were significant using this approach (**Figure 4A**). To select the most robust features, we adopted a multistep screening strategy as in a previous study (28, 29). The LASSO, SVM-RFE, and Boruta algorithms further reduced the number of features to 3, including NMLR, cTn I ≥ 20ULN, and NT-proBNP (**Figures 4B–E**). Based on the above results, we established a nomogram based on NMLR (OR = 1.56, 95% confidence interval [CI]: 1.00−2.43), cTn I ≥ 20ULN (OR = 4.95, 95% CI: 1.57−15.60), and NT-proBNP (OR = 4.77, 95% CI: 1.75−12.97), along with a web-based online risk calculator as shown in **Figure 4F** and **Supplementary Figure 5**. The online tool can be accessed at: https://hebmulzl08.shinyapps.io/icim2/, which allows researchers to calculate the predicted probability and enables them perform model validation using their own cohort with reporting the validation outcomes. The C-index for the prediction nomogram for severe ICI-M was 0.849 (95% CI: 0.767−0.931) and presented as 0.849 (95% CI: 0.763−0.925) after 1000 bootstrapping validation. As shown in **Table 2**, we implemented the DeLong test to quantitatively compare the AUC values of the integrated nomogram and each of the three independent predictive features. All comparisons yielded statistically significant differences (p< 0.05), which supported the advantage of the multivariable nomogram over single-feature prediction alone. We further conducted subgroup sensitivity analysis based on the predicted probability values derived from our model (**Supplementary Figure 6**). The predicted probabilities of severity of ICi-M confirmed the significant risk factors across all subgroups, with the exception of patients receiving non-PD-1 monotherapy. The calibration curve for predicting severe ICI-M showed a relatively good degree of fitting. The decision curve indicated that using this nomogram to predict the severe ICI-M may add more benefits when considering the probability of 0.353 as a decision threshold. As shown on bottom right of **Figure 4G**, the nomogram (SE: 80.0%, SP: 82.9%, PPV: 66.7%, NPV: 90.6%, accuracy: 0.82, F1-score: 0.727) owned a relatively good predictive performance. As displayed in **Supplementary Figure 7A**, the selected severe risk score ≥97.8 provided relatively good risk stratification when exploring its potential value to the 30-day MACE survival, with a significance of p< 0.0001. This threshold of severity risk score also yielded consistent stratification in the subgroup analyses (**Supplementary Figure 7B**).

**Table 2:** Comparison of predictive performance between the nomogram and individual variables in the nomogram.

Variables compared with nomogram	AUC of the variable alone (95% CI)	Z value of DeLong test	P-value of DeLong test
cTnI ≥ 20ULN	0.724 (0.635,0.813)	3.1209	0.002
NT-proBNP	0.780 (0.686,0.875)	1.9777	0.048
NMLR	0.677 (0.551,0.814)	3.5293	<0.001

### 3.6 Single-cell RNA-seq analysis of PBMC samples

**Figure 5:**
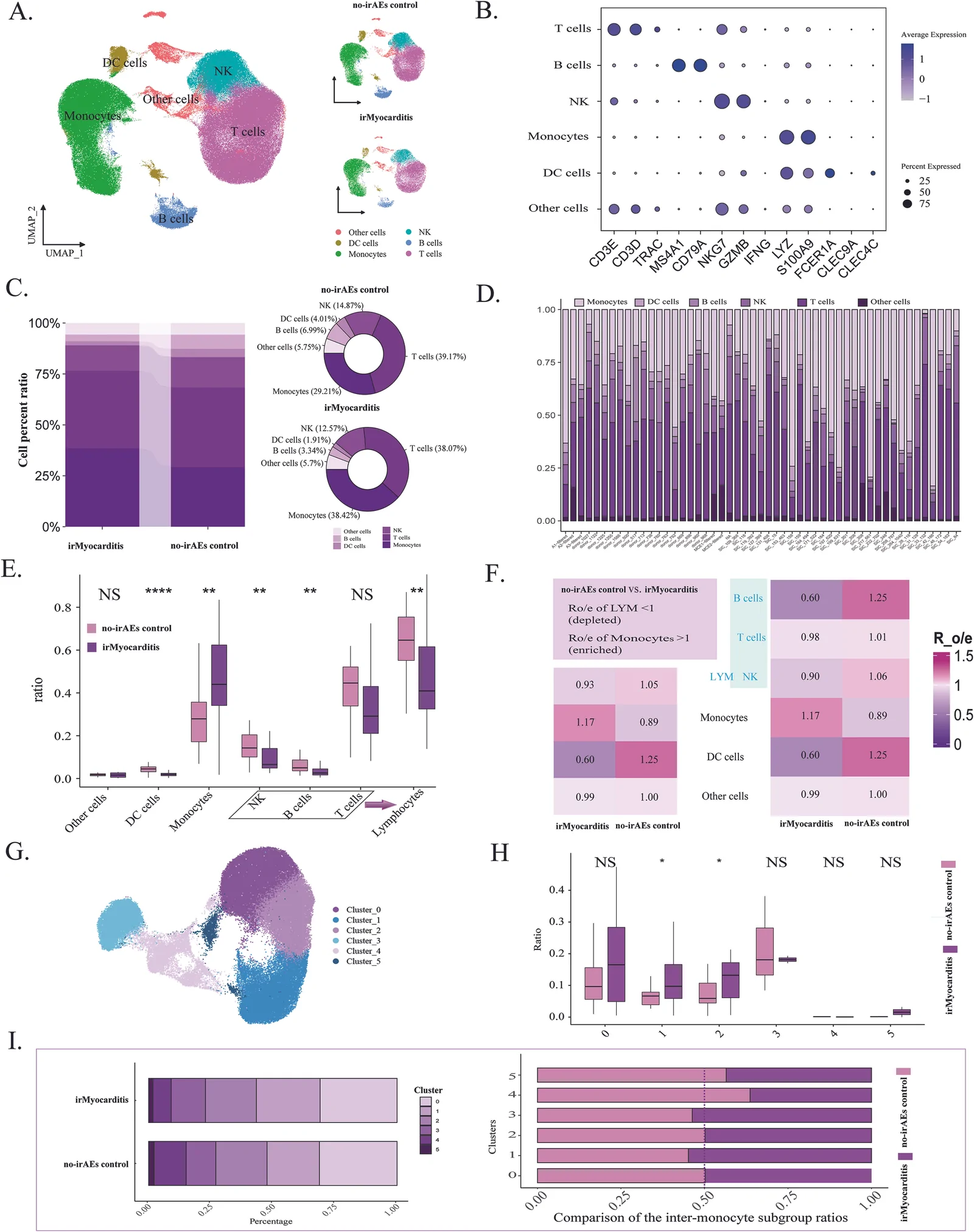
Single-cell analysis reveals monocyte enrichment and lymphocyte depletion in immune checkpoint inhibitor-related myocarditis (ICI-M) patients compared with no-irAEs controls. **(A)** UMAP plot displaying cell populations from PBMCs, dots representing individual cells and colors distinguishing cell types. **(B)** Dot plot showing the expression of characteristic genes across cell types. Dot size represents the proportion of cells expressing the gene, and color intensity reflects the average expression level. Overall **(C)** and sample-level cell-type proportion **(D, E)** comparison of each cell type between the ICI-M and control groups. **(F)** Observed-to-expected ratio (Ro/e) quantifying differences in cellular composition between ICI-M and control groups. **(G)** UMAP plot illustrating re-clustered clusters C0-C5 of monocyte subset. **(H)** Box plots comparing the proportions of clusters C0-C5 of monocyte between ICI-M and control groups. **(I)** Bar chart showing the percentage of monocyte subclusters (Clusters 0-5) among all monocytes in ICI-M versus no-irAEs controls (left), with corresponding inter-group percentage comparison (right). UMAP, Uniform Manifold Approximation and Projection; irAEs, immune-related adverse events; *p< 0.05, **p< 0.01, ****p< 0.0001; NS, not significant.

Following quality control and batch integration, a total of 260,592 single cells (184,709 from GSE228597; 75883 from GSE180045) were retained (**Supplementary Table 5**). After dimensionality reduction analysis, 17 cell clusters were identified. The results of cell type annotation by the ACT tool are displayed in **Supplementary Table 9**. Subsequently, the cells were annotated into 6 groups, including T cells, natural killer (NK) cells, B cells, MOs, DCs and other cells (**Figures 5A, B**). The cell type analysis showed a significant reduction of B cell, NK cell, DCs and LYM proportions and an significant increase of MOs in the ICI-M group in the overall and sample-level proportion analyses (**Figures 5C–E**), compared with the non-irAEs control group. The Ro/e ratio revealed expansion of MOs in the ICI-M group (Ro/e = 1.17) with depletion of LYMs (Ro/e = 0.93), compared to the control group (**Figure 5F**). Due to the marked increase of MOs, we further performed subclustering of the MO population and identified six distinct major subgroups (**Figure 5G**). Notably, both the abundance and proportion of cells in Cluster_1 were significantly elevated, highlighting its potential role in ICI-M (**Figures 5H, I**). Additionally, we explored the association between PBMC sub-cell groups and the ICI-M severity-related trait (fatal and non-fatal). Under a resolution of 0.8, 14 distinct cell clusters (**Supplementary Figure 8A**) were successfully distinguished. The results of cell type annotation by the ACT tool are displayed in **Supplementary Table 9**. Subsequently, the cells were annotated into 6 groups, including T cells, NK cells, B cells, MOs, DCs and other cell types (**Supplementary Figures 8A, B**). The analysis of the proportion of cell types showed that a reduction of B cells, NK cells, DCs, and LYM and an increase of MOs in the fatal ICI-M group in the overall cell population compared with the non-fatal group (**Supplementary Figure 8C**). However, sample-level proportion analysis displayed no significant differences (**Supplementary Figures 8D, E**), The Ro/e ratio revealed expansion of MOs in the fatal ICI-M group (Ro/e = 1.12) with depletion of LYMs (Ro/e = 0.86), compared with the non-fatal group (**Supplementary Figure 8F**).

### 3.7 Monocyte subset analysis between ICI-M and control groups

**Figure 6:**
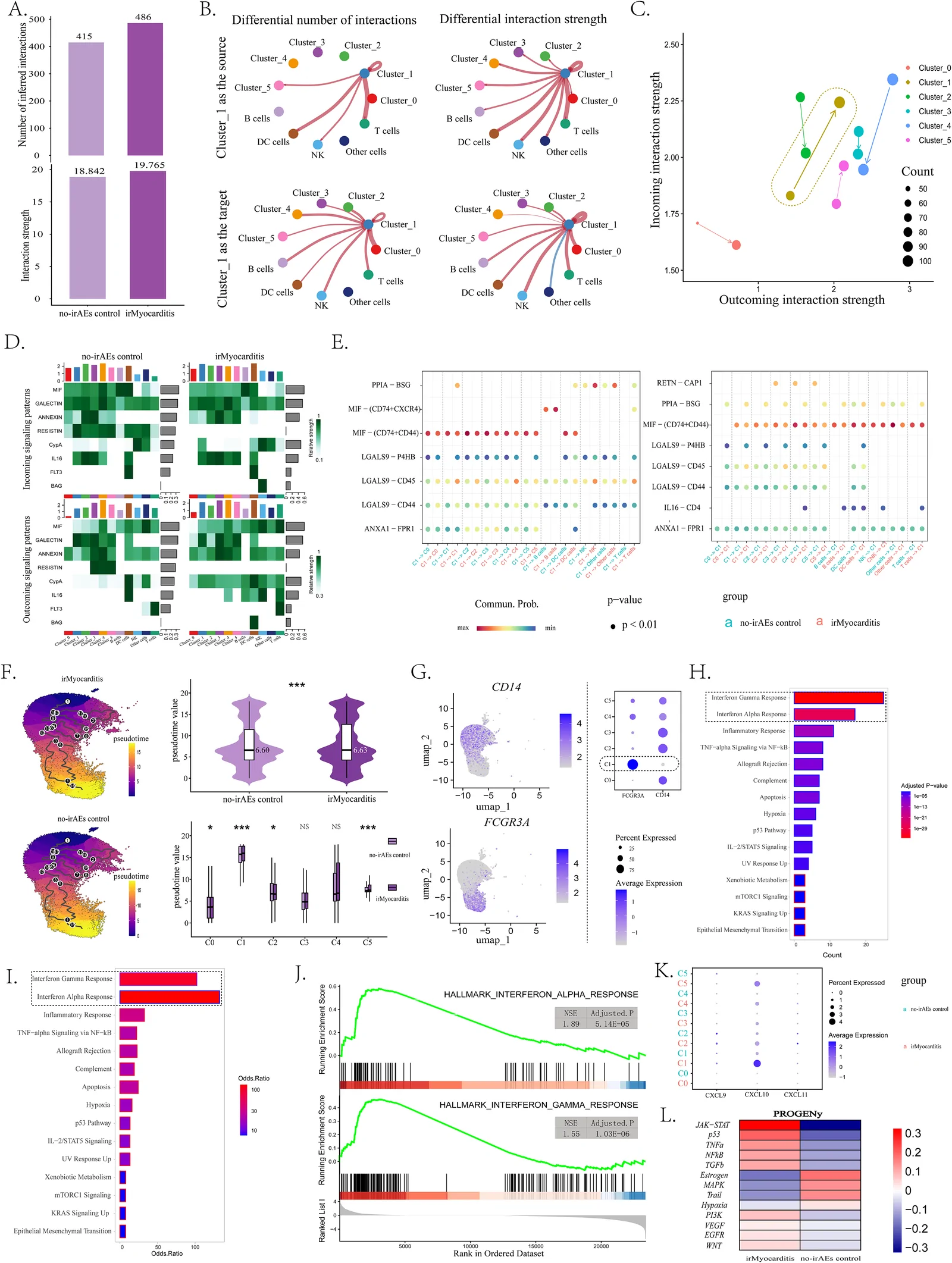
Single-cell dissection of monocyte subclusters reveals an inflammatory and interferon-activated *FCGR3A*+ subset with enhanced cell-cell communication in immune checkpoint inhibitor-related myocarditis (ICI-M) patients. **(A)** Overview of cell-cell communication network among six monocyte subclusters and other immune cell types in ICI-M (right) and non-irAEs control (left) groups. **(B, C)** Differential comparison of overall incoming and outgoing signaling number and strength of cluster_1 monocytes between ICI-M and non-irAEs control groups. **(D)** Comparison of the incoming and outcoming signaling patterns of six monocyte subclusters and other cell types between ICI-M and non-irAEs control groups. **(E)** Heatmap of differential ligand-receptor interaction probabilities, highlighting upregulated pro-inflammatory axes including MIF, PPIA-BSG, and LGALS9 family pairs in ICI-M patients. **(F)** Pseudotime trajectory placing Cluster_1 at the terminal differentiation stage, with higher pseudotime values in the ICI-M group. **(G)** *FCGR3A* expression was significantly elevated in Cluster_1. **(H, I)** Enrichr hallmark pathway analysis showing top 15 enriched pathways (H:ordered by count; I: ordered by odds ratio), with interferon responses as the most prominent. **(J)** GSEA analysis confirming interferon pathway activation in ICI-M. **(K)** The expression of *CXCL9*, *CXCL10*, and *CXCL11* in different monocyte subclusters between the ICI-M and control groups. **(L)** PROGENy analysis revealed JAK-STAT, TNFα, and NF-κB activation, and downregulation of Estrogen, MAPK, and TRAIL pathways in *FCGR3A*+ monocytes among ICI-M patients. Data are presented as mean ± SE. *p< 0.05, ***p< 0.001; NS, not significant.

To clarify the intrinsic heterogeneity and inter-subset crosstalk of MOs between ICI-M and non-irAEs control groups, we first performed comparative CellChat analysis to compare incoming and outgoing signaling patterns of six MO subclusters (**Figures 6A–E**; **Supplementary Figure 9A**). The Cluster_1 of MOs exhibited upregulated communication strength and number specifically in its interactions with T cells and NK cells in ICI-M, suggesting its potential role in modulating the inflammatory microenvironment via enhanced crosstalk (**Figures 6A–D**). Cell–cell communication analysis also revealed enhanced interaction probability of proinflammatory ligand–receptor pairs originating from Cluster_1 MOs in the ICI-M group. As shown in **Figure 6E**, multiple axes, including MIF-related signaling, PPIA-BSG, and LGALS9 family ligand–receptor pairs, displayed markedly elevated interaction probabilities in the ICI-M cohort than the non-irAEs control group. Pseudotemporal trajectory analysis placed Cluster_1 of MOs at the terminal stage of immune cell differentiation, indicating a functionally terminally differentiated phenotype. Moreover, pseudotime quantitative analysis revealed a global- and subcluster-level higher pseudotime value in MOs from the ICI-M group compared with the non-irAEs control group (**Figure 6F**). Based on the differentiation time indicated by the pseudotime trajectory, the gene expression distribution maps with S100A family genes (30) (**Supplementary Figure 9D**), revealed significantly decreased gene expression of *S100A8*, *S100A9*, *S100A10*, and *S100A12*, and increased expression of *S100A11* in the pseudotime trajectory. Subcluster gene expression comparison revealed that Cluster_1 of MOs had significantly higher expression of *FCGR3A* than other clusters (**Figure 6G**). As for S100A family genes, their expression is higher in the ICI-M group, whereas MOs exhibited higher levels than other cell types (**Supplementary Figures 9B, C**). Subsequently, the pathway analysis with Enrichr (dataset: MSigDB Hallmark 2020) identified 15 significantly enriched pathways for ICI-M compared with the non-irAEs control, among which the interferon (IFN) gamma response (Odd Ratio = 102) and interferon alpha response (Odd Ratio = 132) were the most prominently enriched (**Figures 6H, I**). GSEA analysis (**Figure 6J**) further confirmed specific activation of IFN-related pathways in the ICI-M group (NES = 1.89, Adjusted p< 0.01 for interferon alpha response; NES = 1.55 and adjusted p< 0.01 for interferon gamma response). The expression of *CXCL10* was higher in *FCGR3A*+ MOs within the ICI-M group than other subgroups (**Figure 6K**). PROGENy was utilized to compare signaling pathway activity of *FCGR3A*+ MOs between ICI-M and control groups, with pathway activity inferred from the transcriptional levels of pathway-responsive genes. Strong activation was found in the JAK/STAT, TNFα and NFκB signaling pathways in patients with ICI-M, whereas Estrogen, MAPK and TRAIL pathways were downregulated (**Figure 6L**). Additionally, we performed TF activity analysis of *FCGR3A*+ MOs. JUN, IRF7, HES1, FOS, and ETV7 exhibited the top five highest activities among *FCGR3A*+ MOs for ICI-M patients. The pathway enrichment analyses of target genes regulated by the top 5 TFs also identified significant enrichment in IFN related pathways (**Supplementary Figures 9E–G**).

## 4 Discussion

### 4.1 Pharmacovigilance perspective from FAERS

To capture a more comprehensive perspective of ICI-M, we analyzed the FAERS database by identifying signal of ICI-M events. Initially, the FAERS data mining process provided an overview of myocarditis associated with the 8 types of ICIs using different terms. As expected, strong signals of such AEs were detected. Furthermore, the number of reports of myocarditis have gradually increased over the past 5 years, although the increasing number of reports over time may reflect increased use of ICIs, improved detection of ICI-M, reporting bias, or duplicate reports rather than a true increase in myocarditis risk. Nonetheless, the anticipated increase in ICI-M incidence warrants heightened clinical awareness and rigorous investigation into its pathogenesis and management. As for the time from initial therapy to onset, ICI-M typically manifests early after initial ICI therapy, with 76.7% of reported cases occurring within the first 3 months via an AE signal from the pharmacovigilance database VigiBase (6). Additionally, another VigiBase study reported a median time of symptom onset as 30 days (22). Utilizing the recent data from the FAERS database, we identified nearly 80% of the events emerged in the initial 3 months following the ICI therapy, which was similar with the previous studies. In our clinical data, we also observed about 80% of the events emerged in the initial 3 months following the ICI therapy. Hence, we regarded 90-day as a cutoff time point for the occurrence of ICI-M for the following analysis.

### 4.2 Associations between peripheral blood cells and ICI-M

Recently, Zheng et al. (10) demonstrated a significantly lower LYM and a higher MO level in the ICI-M group than an independent non-ICI-M control group. In our study, we did not select an independent control but use a paired rank-sum test to compare the blood cells between before last cycle ICI-therapy and at ICI-M onset within the patients individually. We observed a similar directional change for LYM and MO, which further highlighted their potential role in ICI-M development. Importantly, this observation was obtained from an intrapersonal paired design that inherently adjusted for genetic polymorphisms, cumulative prior therapy, and baseline immune status. Subsequently, we also identified certain associations for blood cells before initial ICI-treatment and at ICI-M onset. Higher pretreatment LYMs and lower pretreatment MOs were observed to be potentially associated with both the 90-day incidence and the severity of ICI-M. Additionally, NE, LYM and MO at ICI-M onset were all significantly different in the severe group compared with the non-severe group, which has also been reported in other studies, corresponding with the significantly decreased tendency for LYM at ICI-M onset, Drobni et al. and Chen et al. (8, 9) also observed a statistically significant decrease for absolute LYM count (ALC) from clinical ICI-M patients at the ICI-M onset. However, with regard to absolute MO count (AMC), few studies have reported differences across ICI-M patients, although a significant increase was observed in this study.

Recent studies have reported a poorer prognosis among patients with severe ICI-M compared to those with non-severe ICI-M, characterized by a shorter median survival time and a higher incidence of MACE (5, 15). This highlights the critical need for early recognition and risk stratification for ICI-M. Moreover, great efforts have been made to explore the biomarkers associated with the occurrence of severe irAEs, and include changes in expression of PD1 and PD–L1 (31), increased expression of *IL1B*, *CXCL8*, and *CXCL2* in MOs and *TNF* in macrophages (32), and a diagnostic index based on Human leukocyte antigen–DRB1 (HLA–DRB1) genotype and PD–L1 expression (33). Nonetheless, limited studies have explored biomarkers for the severity of ICI-M using routine and common clinical indicators. Motivated by the observation that blood cells at ICI-M onset exhibited significant severity-related differences, we performed further analyses using three blood cell-derived ratio indexes, named SIRI, SII and NMLR. Several studies have explored the clinical value of inflammation blood-derived ratio indexes for myocarditis and irAEs cohorts. Guan et al. (11) identified SIRI and SII as risk factors associated with short-term and long-term survival outcomes. Wang et al. (12) revealed an association of SIRI with the severity of acute myocarditis. Post-treatment NMLR was found to be correlated significantly and positively with cancer therapy-related cardiac dysfunction (CTRCD) (34). To evaluate their potential value in predicting severe ICI-M, we systematically analyzed the association between the above three ratio indexes and their change rates (between at onset and before the last ICI receipt) with ICI-M severity. Three indexes at onset, rather than their change rates, were all significantly higher among patients with severe ICI-M. Based on the above evidence, we selected the 3 ratio indexes to explore their potential application for the nomogram development.

### 4.3 Nomogram for predicting ICI-M severity involving blood-derived ratio indexes

In our study, we identified NMLR, cTn I, and NT-proBNP as the key features from the cardiac biomarkers and inflammation ratio indexes to predict severe ICI-M through a multimethod feature-screening strategy (28, 29). The exploratory nomogram was based on the above 3 features achieved a relatively better severity-diagnostic performance compared with individual features, respectively. In previous studies, higher troponin I and NT-proBNP levels have also been observed in patients with severe ICI-M than in those with non-severe ICI-M, which may be inherent to the inflammatory myocyte necrosis of myocarditis (17, 35–38). In the study conducted by Zhou et al. (17), both high-sensitivity Tn I and NT-proBNP were independent predictors of fulminant ICI-M, with AUCs of 0.984 and 0.981, respectively. Nonetheless, the AUCs of these biomarkers in our study were both below 0.80, which may have reflected the confounding effects of the cohort heterogeneity. The inflammatory cascade in acute myocarditis involves coordinated actions of multiple immune lineages NEs, MOs, macrophages, and LYMs (12). Correspondingly, the NMLR integrates aggressive, proinflammatory forces (NEs and MOs) and the regulatory, anti-inflammatory forces (lymphocytes) into a single metric. Among these blood cell types, Carai et al. (39) demonstrated that NEs drive inflammatory cascades in acute myocarditis through their amplified innate immune activity. Patients with severe irAEs also show a significant decrease in lymphocyte counts, which may be related to lymphocytes infiltrated in tissues with irAEs (40). In clinical practice, especially for ICI-M, discordance between cTnI/NT-proBNP levels and the severity of presenting symptoms has frequently been observed. Some patients with markedly elevated biomarkers may exhibit a non-fulminant disease course, whereas others with only modest elevations in these markers develop fulminant myocarditis. This highlights the limitation of using a single biomarker threshold to predict fulminant myocarditis and supports the need for a composite model incorporating both biomarkers and other clinical parameters. Our exploratory analysis indicates that the integration of peripheral blood ratio indexes with conventional cardiac biomarkers offers a potential adjunctive tool for discriminating the need for close clinical monitoring of patients with ICI-M. Given the single-center and small-sample context of our ICI-M cohort, we developed a web-based online risk calculator. This freely accessible online calculator not only allows real-time risk estimation for ICI-M but also allows independent external validation, thereby promoting model transparency and fostering multicenter collaborative improvement. Moreover, we also explored the potential value of the nomogram-based risk score to effect 30-day MACE survival and found a cutoff value of 97.8 may help to identify ICI-M patients who were more inclined to develop MACEs. However, the risk score was developed to classify severe ICI-M, and not to predict MACE. The optimal cutoff using “surv_cutpoint” from the same dataset may introduce “cutpoint optimization bias”. Given the limited sample size and the exploratory nature of this analysis, we underscore that this result should be viewed as preliminary and hypothesis-generating. Prospective studies with adequate statistical power are warranted to validate the prognostic utility of this cutoff and to determine whether it can be integrated into routine clinical decision-making. In the clinical application of our nomogram, we advocate that the nomogram should be interpreted exclusively for patients with ICI-M who have undergone an initial differential diagnostic workup (**Supplementary Tables 3**, **7**) and have been clinically diagnosed with myocarditis according to the 2022 ESC guideline. Furthermore, patients with severe infections or those receiving medications known to alter blood parameters should be excluded, as these conditions may substantially confound the model’s accuracy. Thus, the nomogram is intended for severity stratification rather than initial screening, and its application should be guided by careful clinical judgment.

### 4.4 Immune landscape of PBMC for ICI-M

In our clinical blood samples analysis, we observed significantly decreased ALC and a significantly increased AMC from ICI-M patients when compared with those at the time point before last ICI therapy (non-ICI-M status) to at ICI-M onset. Additionally, such shifts in LYM and MO in our clinical cohort were further corroborated by independent GEO PBMC data showing parallel shifts in LYM and MO proportions. Using 51 PBMC samples from the GEO database (13, 14), the single-cell analysis revealed a significantly decreased proportion of LYM, as well as an significantly increased proportion of MO in the ICI-M group compared with control group, also aligning with the study by Lou et al. (30). As for T cells, another study observed that their proportion in the peripheral blood was significantly decreased in the ICI-M group compared with the control group (41). However, in our analysis, T cells were found to decrease in overall cell analysis among ICI-M patients, but this change was not significant in the sample-level analysis. As for the severity of ICI-M, we used 7 PBMC samples marked with the severity-related traits of fatal and non-fatal. The single-cell analysis revealed expansion of MOs in the fatal ICI-M group with depletion of LYMs, compared with the non-fatal group, although sample-level proportion analysis displayed no significant differences. The overall tendency corresponded with the clinical data and blood cell analysis between severe and non-severe groups. Additionally, we validated the S100A protein family as a potential serum marker for ICI-M as Lou et al. (30) previously demonstrated. In our study, S100A protein family still expressed higher in ICI-M group compared with control group and this upregulation was predominantly attributed to MO subsets. Comparing with their study with 17,729 PBMCs from 3 patients with ICI-M, we combined PBMCs from the public available database and finally involved 10,0712 PBMCs from 19 patients with ICI-M, which constitutes a substantially larger cohort and provides enhanced statistical power to confirm the potential role of S100A family members in peripheral blood.

### 4.5 The potential role of *FCGR3A*+ monocyte subset in ICI-M

In the present study, single-cell analysis of PBMCs from ICI-M patients and non-irAEs controls revealed a marked expansion of MOs, particularly a distinct subcluster characterized by high expression of *FCGR3A* (encoding CD16), a feature typically associated with non-classical MOs. *FCGR3A*+ MOs demonstrated significantly enriched interaction probabilities with T cells and NK cells via several proinflammatory ligand–receptor axes, including MIF-(CD74 + CD44), PPIA-BSG, and LGALS9 family pairs. These findings suggest that *FCGR3A*+ MOs may serve as amplifiers of myocardial inflammation by orchestrating intercellular crosstalk in the peripheral immune compartment. The pseudotime trajectory analysis positioned *FCGR3A*+ MOs at a terminally differentiated stage and the pathway enrichment analysis further revealed a significant activation of both type I (IFN-α) and type II (IFN-γ) interferon responses in the ICI-M group, with higher expression of CXCL10 in the ICI-M group. The pathway enrichment analyses of target genes regulated by the top 5 TFs for *FCGR3A*+ MOs from ICI-M patients also identified significant enrichment in IFN related pathways. IFN-γ is reported markedly elevated in the peripheral blood of patients with ICI-M (42), which is known to promote PD-L1 expression on cardiac cells as a compensatory mechanism to limit excessive CD8+ T cell activity (43). Moreover, Ma et al. (44) linked ICI-M to the expansion of IFN-γ-induced inflammatory macrophages, suggesting IFN-γ blockade as a potential treatment for ICI-M. Emerging clinical observations have suggested that the combination of abatacept (a CTLA4-Ig fusion protein) and ruxolitinib (a Janus kinase inhibitor that blocks IFN-γ/JAK2/STAT1 signaling) is associated with reduced cardiovascular mortality in patients with ICI-M (45). Correspondingly, the JAK/STAT pathway achieved the highest active score in *FCGR3A*+ MOs from ICI-M patients during PROGENy analysis, which may indicate that the improvement role of ruxolitinib on ICI-M survival may be associated with *FCGR3A*+ MOs. As for IFN-α, type I interferon signaling can enhance antigen cross-presentation and promote CD8+ T cell survival, potentially synergizing with IFN-γ to amplify myocardial inflammation. However, the precise role of IFN-α in ICI-M remains less defined, and whether its blockade provides additional benefit beyond IFN-γ inhibition warrants further investigation.

### 4.6 Study limitations

Our study also has some limitations that should be acknowledged. First, the spontaneous reporting system of FAERS, is subject to underreporting, incomplete or low-quality data, duplicate reports, and difficulty controlling confounding factors that may confound the results; thus, causality cannot be definitively established. Therefore, our FAERS findings should be interpreted cautiously, and future studies should employ prospective cohort designs or pharmaco-epidemiological approaches with rigorous confounder adjustment. The temporal trend may reflect multiple non-causal factors, including increased ICI use, heightened awareness, reporting changes, publicity, and duplicate reports, rather than a true rise in myocarditis risk, and may not represent an increase in disease incidence. Second, as a single-center retrospective study with limited cases, this exploratory study inevitably introduces selection bias. The potential circularity of biomarkers, the predominance of gastrointestinal tumors and PD-1 monotherapy may also limit the generalizability of its findings. In the present study, external validation of the model was still not feasible and further multicenter prospective studies are still warranted. However, the developed interactive web-based application may actively facilitate future external validation. Third, endomyocardial biopsy was not routinely used as a primary diagnostic tool in this study, given patient preferences, disease status, and associated procedural risks. Critical status may result in type 2 myocardial injury, which may increase the risk of misclassification bias. However, diagnosis was made by two cardio-oncology specialists, and a detailed differential diagnosis was performed, according to the 2022 ESC Guidelines on cardio-oncology to reduce the bias as much as possible. Fourth, as for the single-cell analysis, although the cell numbers were large (>260,000), the results should be considered mainly descriptive and based on public datasets. Further investigations regarding the in-depth mechanisms remain to be elucidated through experimental confirmation. For the severity analysis, only two fatal samples were available, which limits generalizability of the findings and underscores the need for further validation in larger cohorts. The lack of pre-onset single-cell samples precludes determination of whether the observed blood signatures precede ICI-M, limiting validation of their predictive utility.

## 5 Conclusion

This study systematically characterized the pharmacovigilance features of patients with ICI-M and identified strong and early-onset safety signals for ICI-M across all major ICIs, underscoring the need for heightened clinical vigilance during the initial months of treatment. Clinically, we identified NMLR, cTnI, and NT-proBNP as key predictors of ICI-M severity and developed a nomogram with a relatively good predictive accuracy and clinical utility. The immune landscape insights may inform future therapeutic strategies in ICI-M, especially for INF-related pathways of *FCGR3A*+ MOs. However, future external and multicenter prospective studies are warranted to validate the nomogram, and experimental confirmation is also required for the single-cell analysis results.

## Data Availability

The original contributions presented in the study are included in the article/**Supplementary Material**. Further inquiries can be directed to the corresponding author.

## Glossary

ICI-M: Immune checkpoint inhibitor-related myocarditis
FAERS: FDA Adverse Event Reporting System
ROC: Receiver operating characteristic
PBMC: Peripheral blood mononuclear cell
AUC: Area under curve
CTLA-4: Cytotoxic T-lymphocyte-associated protein 4
PD-1: Programmed death 1
PD-L1: Programmed death-ligand 1
irAEs: Immune-related adverse events
LVEF: Left Ventricular Ejection Fraction
ECG: Electrocardiogram
SII: Systemic Immune-Inflammation Index
PTs: Preferred terms
ROR: Reporting odds ratio
PRR: Proportional reporting ratio
EBGM: Empirical Bayesian geometric mean
IC: Information component
AEs: Adverse events
ESC: European Society of Cardiology
CTCAE: Common Terminology Criteria for Adverse Events
MACEs: Major adverse cardiovascular events
LR: Logistic regression
SVM-RFE: Support Vector Machine - Recursive Feature Elimination
AUCs: Area under the receiver operating characteristic curves
DCA: Decision curve analysis
SE: Sensitivity
SP: Specificity
PPV: Positive predictive value
NPV: Negative predictive value
GEO: Gene Expression Omnibus
UMI: Unique Molecular Identifier
PCA: Principal component analysis
UMAP: Uniform Manifold Approximation and Projection
BMI: Body Mass Index
OR: Odds ratio
HLA-DRB1: Human leukocyte antigen-DRB1
EMB: Endomyocardial Biopsy
ESC: European Society of Cardiology
