# Supplementary material for: Peripheral blood cells and immune checkpoint inhibitor-associated myocarditis: a retrospective clinical study with pharmacovigilance and single-cell analysis
Source: Front Immunol. 2026 Aug 26;17:1834913. doi: 10.3389/fimmu.2026.1834913 (PMC13553236; doi:10.3389/fimmu.2026.1834913)
Supplement: Supplementary file 1 [file DataSheet1.zip › 1834913_SupMaterial/Supplementary Figures/Supplementary figure legends.docx]

**Legends of the supplementary figures**

**Figure S1** Supplementary pharmacovigilance analysis of ICI-M defined by Gougis et al. in FAERS database. (A) Disproportionality measures (ROR, IC) for 8 types of ICIs. (B) Time-to-onset distribution of ICI-M using the broader case definition by Gougis et al. (C) Onset time and its cumulative percentage of ICI-M reports over time since ICI initiation. ICI: Immune checkpoint inhibitors; ICI-M: ICI-associated myocarditis; ROR:reporting odds ratio; IC: information component.

**Figure S2** Supplementary clinical characteristics of the ICI-M cohort. (A) Comorbidity distribution between severe and non-severe ICI-M groups. (B) Time from initial ICI therapy to ICI-M onset in the study cohort. (C) Distribution of ICI types and cancer types among enrolled ICI-M patients. (D) Distribution of major clinical criterion for fulminant myocarditis among 30 severe ICI-M patients. (E) Distribution of concomitant non-myocarditis irAEs among enrolled ICI-M patients. (F) Distribution of clinical symptoms among enrolled ICI-M patients. ICI: Immune checkpoint inhibitors; ICI-M: ICI-associated myocarditis; irAEs: immune-related adverse events.

**Figure S3** Association between peripheral blood cell counts and ICI-M. (A) Paired comparison of lymphocyte count (ALC), monocyte count (AMC), absolute platelet count (APC) and LY to MO ratio (LMR) before last ICI therapy and at ICI-M onset across different patient subgroups. (B) Comparison of ALC, AMC, APC and absolute neutrophil count (ANC) before initial ICI therapy between patients who developed ICI-M within 90 days and those who did not. (C) Comparison of ALC, AMC, APC and ANC before initial ICI therapy between severe and non-severe ICI-M groups. (D) Comparison of ALC, AMC, APC and ANC at ICI-M onset between severe and non-severe ICI-M groups. ICI: Immune checkpoint inhibitors; ICI-M: ICI-associated myocarditis.

**Figure S4** Subgroup analysis of inflammatory ratio indexes and ICI-M severity. (A) Box plots comparing SIRI, SII, and NMLR at onset between severe and non-severe groups in patients without other irAEs. (B) SIRI, SII, and NMLR before last ICI therapy between severe and non-severe groups in patients receiving >1 cycle of ICI. (C) Association of △SIRI, △SII, and △NMLR with severity after excluding patients with only one ICI cycle and those with other irAEs. ICI: Immune checkpoint inhibitors; ICI-M: ICI-associated myocarditis; irAEs: immune-related adverse events; SIRI: Systemic Inflammation Response Index; SII: Systemic Immune-Inflammation Index; NMLR: (neutrophil + monocyte) to lymphocyte ratio .

**Figure S5** Web-based online risk calculator for severe ICI-M prediction.

**Figure S6** Subgroup sensitivity analysis based on the predicted probability values derived from our nomogram.

**Figure S7** Risk stratification for 30-day MACE based on nomogram-derived severity score. (A) Kaplan-Meier curves for 30-day MACE-free survival in high-risk v.s. low-risk groups (cutoff: 97.8). (B) Subgroup analyses of 30-day MACE risk stratified by the severity score across different clinical subgroups. MACE: major adverse cardiovascular events.

**Figure S8** Single-cell immune profile for peripheral blood mononuclear cell (PBMC) from the two fatal and 7 non-fatal samples of immune checkpoint inhibitor-related myocarditis (ICI-M). (A) Uniform Manifold Approximation and Projection (UMAP) dimensionality reduction visualization based on gene expression profiles, with each dot representing an individual cell and colors indicating major cell types. (B) Dot plot showing the expression of characteristic genes across cell types. Dot size represents the proportion of cells expressing the gene, and color intensity reflects the average expression level. Overall (C) and sample-level cell-type proportion (D and E) comparison of each cell type between the ICI-M and control groups. (F) Observed-to-expected ratio (Ro/e) quantifying differences in cellular composition between the fatal ICI-M and non-fatal groups.

**Figure S9** (A) Differential comparison of overall incoming and outgoing signaling strength of 5 monocyte subclusters between ICI-M and non-irAEs control groups. (B) Dot plot showing the expression of S100A protein family genes among ICI-M and non-irAEs control groups. Dot size corresponds to proportion of cells within the group expressing each gene, and dot colour corresponds to expression level. (C) Dot plot showing the expression of S100A protein family genes in each cell population. (D) Pseudotime trajectory of the gene expression distribution map with 5 significantly differential S100A protein family genes. (E) Regulon Specificity Scores (RSS) of key transcription factors in the *FCGR3A*+ monocytes within the ICI-M group (F) Dot plot showing the top five regulons identified in the subtype *FCGR3A*+ monocytes using pySCENIC analysis. (G) Bar plot displaying pathway enrichment analysis of target genes regulated by the top five transcription factors in the *FCGR3A*+ monocytes within the ICI-M group
