# Supplementary material for: Peripheral blood cells and immune checkpoint inhibitor-associated myocarditis: a retrospective clinical study with pharmacovigilance and single-cell analysis
Source: Front Immunol. 2026 Aug 26;17:1834913. doi: 10.3389/fimmu.2026.1834913 (PMC13553236; doi:10.3389/fimmu.2026.1834913)
Supplement: Supplementary file 1 [file DataSheet1.zip › 1834913_SupMaterial/Supplementary Tables/Table 2.docx]

**Table S2. Definitions for Immune Checkpoint Inhibitor-Related Myocarditis**

| **① 2022 European Society of Cardiology (ESC) Guidelines on cardio-oncology** | |
| --- | --- |
| **Pathohistological diagnosis**  Multifocal inflammatory cell infiltrates with overt cardiomyocyte loss by light microscopy | |
| **Clinical diagnosis** :  A troponin elevation (new, or significant change from baseline) with 1 major criterion or a troponin elevation (new, or significant change from baseline) with 2 minor criteria after exclusion of acute coronary syndrome or acute infectious myocarditis based on clinical suspicion  **Major Criterion**  • CMR diagnostic for acute myocarditis (modified Lake Louise criteria)  **Minor Criteria**  • Clinical syndrome (including any one of the following: fatigue, myalgias, chest pain, diplopia, ptosis,  shortness of breath, orthopnoea, lower-extremity oedema, palpitations, light-headedness/dizziness,  syncope, muscle weakness, cardiogenic shock)  • Ventricular arrhythmia (including cardiac arrest) and/or new conduction system disease  • Decline in LV systolic function, with or without regional wall motion abnormalities in a non-Takotsubo  pattern  • Other immune-related adverse events, particularly myositis, myopathy, myasthenia gravis  • Suggestive CMR | |
| **② Bonaca et al.** | |
| **Definitive** | • Pathology  OR  • Diagnostic CMR + syndrome + biomarker or ECG  OR  • Echo WMA+ syndrome + biomarker þ+ ECG + negative angiography |
| **Probable** | • Diagnostic CMR (no syndrome, ECG, biomarker)  OR  • Suggestive CMR with either syndrome, ECG or biomarker  OR  • Echo WMA and syndrome (with either biomarker or ECG)  OR  • Syndrome with PET scan evidence and no alternative diagnosis |
| **Possible** | • Suggestive CMR with no syndrome, ECG or biomarker  OR  • Echo WMA with syndrome or ECG only  OR  • Elevated biomarker with syndrome or ECG and no alternative diagnosis |
