# Supplementary material for: Peripheral blood cells and immune checkpoint inhibitor-associated myocarditis: a retrospective clinical study with pharmacovigilance and single-cell analysis
Source: Front Immunol. 2026 Aug 26;17:1834913. doi: 10.3389/fimmu.2026.1834913 (PMC13553236; doi:10.3389/fimmu.2026.1834913)
Supplement: Supplementary file 1 [file DataSheet1.zip › 1834913_SupMaterial/Supplementary Tables/Table S1.docx]

**Table S1** The principles of disproportionate measurement and the criteria for signal detection.

| Method | Calculation formula | Criteria |
| --- | --- | --- |
| ROR | $ROR=\frac{a / c}{b / d}$ | a ≥ 3  95%CI (lower limit) > 1 |
|  | $SE(lnROR)=\sqrt{\frac{1}{a}+\frac{1}{b}+\frac{1}{c}+\frac{1}{d}}$ |  |
|  | $95\%CI= e^{\ln\left( ROR \right)\pm1.96se}$ |  |
| PRR | $PRR=\frac{a / (a+b)}{c / (c+d)}$ | a ≥ 3  PRR ≥ 2  95%CI (lower limit) > 1 |
|  | $SE(lnPRR)=\sqrt{\frac{1}{a}-\frac{1}{a+b}+\frac{1}{c}-\frac{1}{c+d}}$ |  |
|  | $95\%CI= e^{\ln\left( PRR \right)\pm1.96se}$ |  |
|  | $\chi2 =\frac{{(ad-bc)}^{2}(a+b+c+d)}{( a+b)(a+c)(c+d)(b+d)}$ | a ≥ 3  PRR ≥ 2  $\chi2\geq4$ |
| BCPNN | IC=${log}_{2}\frac{p(x,y)}{p(x)p(y)}={log}_{2}\frac{a(a+b+c+d)}{(a+b)(a+c)}$ | IC025>0 |
|  | E(IC)=${log}_{2}\frac{(a+\gamma11)(a+b+c+d+\alpha)(a+b+c+d+\beta)}{（a+b+c+d+\gamma）(a+b+\alpha1)(a+c+\beta1)}$ |  |
|  | $V\left( IC \right)=\frac{1}{{(ln2)}^{2}}\{\left[ \frac{\left( a+b+c+d \right)-a+\gamma-\gamma11}{\left( a+\gamma11 \right)\left( 1+a+b+c+d+\gamma\right)} \right]+\left[ \frac{\left( a+b+c+d \right)-\left( a+b \right)+\alpha-\alpha1}{\left( a+b+\alpha1 \right)\left( 1+a+b+c+d+\alpha\right)} \right]+\left[ \frac{\left( a+b+c+d \right)-\left( a+c \right)+\beta-\beta1}{\left( a+c+\beta1 \right)\left( 1+a+b+c+d+\beta\right)} \right]\}$ |  |
|  | $\gamma=\gamma11\frac{(a+b+c+d+\alpha)(a+b+c+d+\beta)}{(a+b+\alpha1)(a+c+\beta1)}$ |  |
|  | *IC-2SD=E(IC)-2*$\sqrt{V(IC)}$  $\alpha1=\beta1=1；\alpha=\beta=2；\gamma11=1$ |  |
| EBGM | $EBGM=\frac{a(a+b+c+d)}{\left( a+c \right)(a+b)}$ | EBGM05>2 |
|  | $SE(lnEBGM)=\sqrt{\frac{1}{a}+\frac{1}{b}+\frac{1}{c}+\frac{1}{d}}$ |  |
|  |  |  |
|  | $95\%CI= e^{\ln\left( EBGM \right)\pm1.96se}$ |  |

Note: “a” as the number of cases with target events reported with the drug of interest; “c” as the number of cases with target events reported with the comparator; “b” as the number of all other events reported with the drug of interest; and “d” as the number of all other events reported with a comparator.
