# Supplementary material for: Peripheral blood cells and immune checkpoint inhibitor-associated myocarditis: a retrospective clinical study with pharmacovigilance and single-cell analysis
Source: Front Immunol. 2026 Aug 26;17:1834913. doi: 10.3389/fimmu.2026.1834913 (PMC13553236; doi:10.3389/fimmu.2026.1834913)
Supplement: Supplementary file 1 [file DataSheet1.zip › 1834913_SupMaterial/Supplementary Tables/Table S3.docx]

**Table S3** Differential Diagnosis between immune checkpoint inhibitor-related Myocarditis vs. Chemotherapy / Radiotherapy / Targeted Therapy Cardiac Toxicity

| **Comparison Item** | **ICI Myocarditis** | **Chemotherapy-Related Cardiac Toxicity** | **Radiotherapy-Related Cardiac Toxicity** | **Targeted Therapy Cardiac Toxicity** |
| --- | --- | --- | --- | --- |
| **Onset Time** | Median 30–60 days after ICI initiation; most within first 3 months; acute fulminant onset over days | Anthracyclines: cumulative dose-dependent, weeks to years post-chemo; Taxane/FU: acute during infusion; slow progressive heart failure | Manifest months to years after thoracic radiation; can present acutely if combined with ICI/chemo | HER2 inhibitors: within 1–6 months; TKIs/anti-angiogenics: during treatment cycles; onset usually subacute |
| **Key Clinical Manifestations** | Rapid dyspnea, chest pain, palpitations, syncope; high risk of complete AV block, sustained ventricular tachycardia; frequently accompanied by other irAEs (pneumonitis, thyroiditis, enteritis, rash); high risk of cardiogenic shock | Gradual exercise intolerance, orthopnea, peripheral edema; atrial arrhythmias common; severe high-grade conduction block rare; no systemic immune inflammatory symptoms | Chronic dyspnea, constrictive pericarditis symptoms, recurrent pleural effusion; late valvular disease, coronary stenosis; minimal acute arrhythmia crisis | Asymptomatic LVEF drop most common; mild exertional dyspnea; anti-VEGF agents may trigger hypertension/heart failure; rarely malignant ventricular arrhythmias |
| **Medication Exposure Background** | PD-1/PD-L1 monotherapy, dual ICI, ICI combined with chemo/radiotherapy | Prior anthracyclines, taxanes, fluoropyrimidines; high cumulative anthracycline dose | Thoracic mediastinal/chest wall radiation; often combined with anthracyclines or immunotherapy | Trastuzumab, pertuzumab, small-molecule HER2 TKIs; anti-VEGF/VEGFR agents |
| **Cardiac Biomarkers (cTn, NT-proBNP)** | Troponin sharply elevated with very high peak; NT-proBNP elevated synchronously; markers fluctuate rapidly | Troponin mildly elevated or normal; persistent isolated NT-proBNP elevation dominant | Troponin normal in stable chronic phase; mild troponin rise only during acute pericarditis | Troponin mostly normal; mild NT-proBNP elevation when LVEF declines |
| **Systemic Inflammatory Markers (CRP, IL-6)** | Markedly elevated CRP, IL-6, soluble IL-2 receptor | CRP slightly elevated or normal; no persistent inflammatory surge | Mild CRP elevation only during acute pericardial inflammation; baseline normal | Inflammatory markers within normal range |
| **ECG Characteristics** | Widespread diffuse ST-T changes; frequent high-grade AV block, bundle branch block, sustained ventricular tachycardia | Nonspecific ST-T changes; atrial fibrillation/premature atrial beats predominate; severe conduction block uncommon | Chronic T-wave inversion; low voltage; no acute malignant arrhythmia bursts | Mostly normal ECG or minor ST-T depression; rare severe conduction defects |
| **Echocardiogram Features** | LVEF variable (normal to severely reduced); diffuse myocardial edema, markedly impaired GLS; frequent pericardial effusion | Persistent progressive LVEF decline; no obvious myocardial edema; pericardial effusion rare | Pericardial thickening/effusion, restrictive filling pattern; late valvular regurgitation; regional wall motion defects from radiation coronary injury | Isolated reduced LVEF; no myocardial edema; no significant pericardial effusion |
| **Cardiac MRI (CMR) Manifestations** | Classic triad: diffuse myocardial T2 edema, subepicardial/mid-wall LGE, pericardial enhancement/effusion | Minimal or absent T2 edema; scattered tiny subendocardial LGE; no pericardial inflammation | Pericardial thickening/enhancement; focal fibrosis at radiation field; no diffuse myocardial edema | No myocardial edema; absent or minimal LGE; normal pericardium |
| **Core First-Line Treatment** | High-dose glucocorticoids; hold/permanently discontinue ICI; add IVIG/immunosuppressants for critical cases | Standard guideline-directed heart failure therapy; suspend cardiotoxic chemotherapy | Diuretics, anti-inflammatory drugs for pericarditis; heart failure medications; surgical pericardiectomy for constrictive pericarditis | Hold targeted agents; standard heart failure therapy; adjust blood pressure for anti-angiogenic toxicity |
| **Treatment Response Feature** | Troponin, arrhythmia and inflammatory markers improve rapidly within days after steroid treatment | No response to steroids; cardiac function improves slowly with long-term heart failure drugs | Steroids only relieve acute pericardial inflammation; chronic radiation fibrosis responds poorly to immunosuppression | LVEF gradually recovers after drug interruption; steroids have no therapeutic effect |
